# Vaccination

**Published:** 1907-04

**Authors:** 


### VACCINATION.

Dock believes that recent epidemics of smallpox show conclusively the need of more vaccination. He says that what is needed is not statutory compulsion, but an organized and scientific procedure that shall have the confidence and support of a large majority of the people and that shall have no weak spots in any part. The present methods make certain a high ratio of smallpox cases with an unduly low protection to the individual. There is nothing, he asserts, in the fundamental law of the land to prevent the passage of safe and efficient vaccination laws, which should aim at a widespread protection by vaccination and revaccination. The operation itself should be a matter of permanent record, and a certificate from an authorized official should be proof of the vaccination of each individual. The operators should be trained for their work, familiar with the vaccination laws, and bound to follow them. Dock would control the manufacture of vaccine by competent experts. He says that vaccination should be done at fixed times of the year when epidemic diseases are not most prevalent, in places appointed and equipped for the purpose; the individual should be examined after the operation at a time fixed by the regulations, or at once on suspicion of complication. He would permit private vaccination only under special conditions with revision of the result by a competent health officer.—American Jour. Med. Sciences, February, 1907.

